# Surgical versus non-surgical interventions for adolescent idiopathic scoliosis: a Cochrane review protocol

**DOI:** 10.1186/1748-7161-8-S2-O12

**Published:** 2013-09-18

**Authors:** Josette Bettany-Saltikov, Hans Rudolph Weiss, Nachiappan Chockalingam, Razvan Taranu, Shreya Srinivas, Sally Stapley, Julie Hogg, Victoria Whittaker, Raman Kalyan

**Affiliations:** 1 Teesside University, Middlesbrough, UK

## Background

The main aims of all clinical interventions in the treatment of adolescent idiopathic scoliosis (AIS) are to limit curve progression, restore trunk balance and prevent long-term consequences of the deformity. Two separate Cochrane reviews have already reviewed the effects of non-surgical interventions (Negrini, 2010 and Romano, 2012). A further scoping search identified four systematic reviews, however full methodological appraisals within these reviews were very limited (Weiss, 2008), indicating the need for a high-quality Cochrane review focusing on surgical interventions.

## Purpose

The goal of this study is to evaluate surgical versus non-surgical interventions in patients with AIS, both in the short- and long-term.

## Methods

The primary analysis will combine the results of RCTs and QRCTs. We will also include non-randomised prospective studies with a control group since there is a paucity of RCTs in this area. The review will include all types of instrumented surgery with fusion, aimed at providing curve correction and spine stabilisation. Studies describing non-instrumented spinal correction and fusion will be excluded because it has been shown that they do not provide any better outcome than untreated scoliosis (Lonstein, 2006). All outcomes will be measured in the immediate post-operative, short-term, within two years and long-term results into old age. Primary outcomes will include trunk balance, Cobb angle, angle of trunk rotation; number of patients progressed by more than 5° Cobb as well as topographical and psychological outcomes. Searches of COCHRANE, MEDLINE, EMBASE, CINAHL, PsycInfo and PEDro will be conducted. Reference lists of ongoing trials, grey and non-English literature will be screened. Two review authors will screen search results by reading titles and abstracts. Relevant studies will be obtained in full text and independently assessed for inclusion. Risk of bias for RCTs and QRCTs will be assessed using the 12 criteria recommended by the Cochrane Back Review Group and the Newcastle-Ottawa Scale will be used to assess NR trials.

## Results

If a meta-analysis is not possible, results will be described qualitatively and overall quality of evidence for each outcome will be assessed.

## Conclusions and discussion

This protocol has been submitted to the CBRG for review and publication is anticipated later this year.
